# Shaping immunity: Genetic diversity as a key resource for crop protection

**DOI:** 10.1371/journal.ppat.1013892

**Published:** 2026-02-05

**Authors:** Amit Fenn, Samara Mireza Correia de Lemos, Thomas Lahaye, Claude Becker, Ralph Hückelhoven, Nadia Kamal

**Affiliations:** 1 Helmholtz Munich—German Research Center for Environmental Health, Neuherberg, Germany; 2 Technical University of Munich, TUM School of Life Sciences, Freising, Germany; 3 Center for Plant Molecular Biology, University of Tübingen, Tübingen, Germany; 4 Faculty of Biology, Ludwig-Maximilians-Universität, Martinsried, Germany; University of Tübingen: Eberhard Karls Universitat Tubingen, GERMANY

Expanding genetic diversity for disease resistance is crucial to resilient agriculture, particularly as climate change accelerates the emergence and spread of diseases. While crop breeding has achieved tremendous progress in reducing susceptibility, domestication and intensive selection have gradually narrowed the diversity accessible to breeders [1]. Modern genomics now enables the systematic exploration of hidden immune diversity from landraces, crop wild relatives, and underutilised gene pools.

## Genetic diversity underpins crop disease resistance

Genetic diversity provides the raw material for adaptation to evolving pathogens and variable climate. Genomic approaches such as GWAS, QTL, and major resistance (*R*) gene mapping, as well as comparative sequencing, now systematically identify resistance loci across major crops, uncovering alleles that confer protection against fungal, bacterial, viral and nematode pathogens [2–5].

Crop wild relatives, shaped by natural selection, remain particularly rich in untapped *R* genes and QTL that have repeatedly reinforced disease resistance in domesticated lines. Examples include wild progenitors in cereals and wild Solanum species that contribute resistance to rusts, blight and other major diseases [6–10]. In addition to crop wild relatives, landraces shaped by long-term cultivation under local conditions harbour genetic adaptations to regional stresses, including pathogen resistance genes such as *Lr34* for broad fungal resistance [11] and *Pm3* alleles for race-specific powdery mildew resistance in wheat [12].

Together, these examples highlight how diversity beyond modern elite breeding pools continues to sustain modern breeding. Wild and traditional germplasm capture resistance architectures, narrowed through domestication, which can now be systematically mapped, compared and reintroduced into elite lines using genomics.

## Pan-genomics uncovers hidden structural variants associated with immunity

While natural populations, landraces and wild relatives harbour valuable disease resistance alleles, much of this diversity remains invisible to scientists and breeders. Structural variants, gene copy-number changes and presence–absence variation often underlie adaptive immune traits but are difficult to map when relying on a single reference genome. Pan-genomics overcomes this limitation by capturing the full complement of genes and structural variants across multiple individuals, enabling systematic exploitation of immune-gene diversity.

**Fig 1:**
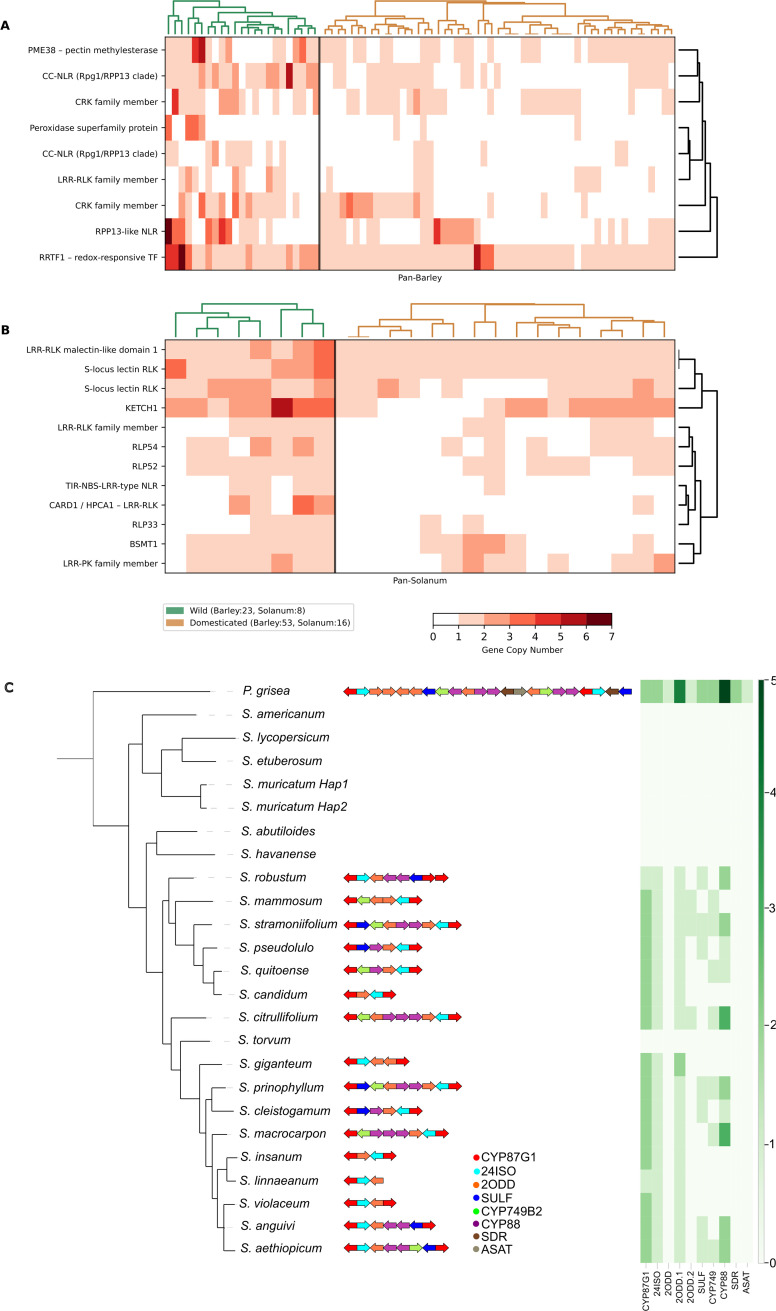
Comparative genomic analysis of defence-related genes in wild and domesticated barley (A) and Solanum (B). Abbreviations are listed in S1 Table. **(C) Biosynthetic gene clusters in Solanum species**. The phylogenetic tree shows species relationships. In the centre, arrows represent co-located genes with standardised lengths and are colour-coded by gene class. The heatmap summarises copy numbers per gene family, considering only genes clustered on the same chromosome. Hierarchical orthogroups (HOGs) were inferred with OrthoFinder [35]. CYP: cytochrome P450 monooxygenases; 24ISO: sterol Δ^24^-isomerase; ODD: 2-oxoglutarate-dependent dioxygenase; ST: sulfotransferase; SDR: short-chain dehydrogenases/reductases; ASAT: acylsugar acyltransferase.

Recent plant pan-genome studies reveal pervasive structural variation (SV) and substantial gene-content turnover across lineages, reshaping our understanding of immune loci and other agronomic traits. In barley, long-read assemblies from 76 wild and domesticated accessions identified loci with CNV, including novel alleles at the *Mla* powdery mildew resistance locus, highlighting the functional importance of SV-rich regions [13]. Building on this resource, our analysis shows that wild barley (*H*ordeum *vulgare ssp. spontaneum*) harbours expanded clusters of immune receptor genes and defence regulators compared to cultivated lines, including canonical nucleotide-binding leucine-rich repeat (NLR) families and genes involved in reactive oxygen species-signalling, cell-wall modification, and transcriptional regulation [14–18] (Fig 1a and S1 Table).

Pan-genomes now also extend beyond single species to entire genera, enabling phylogenetic surveys of immune-gene diversity at larger scales. Using the *Solanum* genus-wide pan-genome [19]*,* we surveyed immune-gene diversity across wild and domesticated lineages. The analysis revealed lineage-specific expansions in receptor-like kinases and NLR families, including members associated with pathogen-associated molecular pattern perception and effector-triggered immunity [20,21] (Fig 1b and S1 Table). These cross-species comparisons demonstrate that immune-gene diversification follows distinct evolutionary trajectories in wild versus cultivated species, reflecting ecological pressures and domestication-driven constraints.

These results underscore wild germplasm as a rich source of immune-gene diversity, where SV and gene-family expansion reveal previously hidden layers of defence complexity. The uncovered loci provide valuable candidates for cloning, association mapping and deployment in resistance breeding.

## Metabolic gene clusters represent an underexplored resource for plant defence

Beyond canonical R genes, biosynthetic gene clusters (BGCs) for specialised metabolites contribute to plant immunity by producing antimicrobials and modulating defence [22]. Their high structural diversity makes them a rich yet underexplored source of resistance, as exemplified by the benzoxazinoid cluster in maize [23]. Recent advances in long-read sequencing and pan-genomics now allow high-resolution analysis of these complex loci.

A compelling example is the withanolide BGC in *Physalis grisea* [24]. Withanolides—steroidal lactones predominantly found in *Solanaceae* but also in *Dioscoreaceae, Asteraceae* and some soft corals [25]—are encoded by a cluster, also present in *W. somnifera [*26*]*. Using the genus-wide Solanum pan-genome [19], we found that this BGC was absent from close relatives of cultivated potato and tomato (Fig 1c) and from several glycoalkaloid-producing Solanum species. This pattern suggests a lineage-specificity of the withanolide pathway within *Solanum*.

Several Solanum species harbour partial withanolide BGCs—typically five of the six validated enzymes (Fig 1c). Similar partial clusters occur in glycoalkaloid-producing taxa, challenging the notion that withanolide and glycoalkaloid pathways are mutually exclusive [27]. We also detected partial clusters in 13 other species, including four glycoalkaloid-producers.

## From classical breeding to precision resistance engineering

Resistance breeding largely relied on wide crosses/backcrossing to introgress resistance, but linkage drag and reproductive barriers made this slow. Today, genomic selection, chromosome engineering and genome editing compress that timeline. Genetic engineering has expanded the toolbox for resistance breeding by enabling targeted manipulation of host genes, pathogen-recognition systems and specialised metabolic pathways. Yet, its impact on large-scale crop improvement remains limited, partly due to regulatory and adoption barriers for transgenic approaches, as well as the still-restricted deployment of engineered traits in agriculture [28]. Consequently, durable progress continues to arise primarily from breeding programmes that combine genomic information with phenotypic selection.

In our view, the greatest potential for accelerating resistance breeding lies in integrating genetic engineering into established breeding pipelines. Gene editing can optimise natural R alleles identified through GWAS or QTL mapping, speed introgression from wild relatives by reducing linkage drag and precisely modify susceptibility genes revealed by comparative genomics. Pan-genomic resources delineate resistance haplotypes to edit or introgress, and genomic selection then prioritises lines that both carry these alleles and show high whole-genome breeding values, thereby enabling efficient stacking of complementary resistance mechanisms—for example, *Agropyron cristatum* segments conferring leaf rust resistance in wheat [29]. Together, these integrated approaches unite natural diversity with molecular precision, enabling faster development of cultivars with durable, broad-spectrum disease resistance.

Modern genome engineering is increasingly applied to BGCs involved in specialised defence compound production. *In planta* studies have demonstrated the efficient multiplexed activation of gene families, thereby boosting metabolite levels across generations [30]. For clusters absent or fragmented in elite lines, synthetic biology strategies such as *de novo* assembly, promoter engineering, or multiplex CRISPR editing enable reconstruction of functional pathways and introduction of novel metabolic traits [31]. As multi-omics resources reveal candidate BGCs with antimicrobial and defence potential, genome editing offers a precise and versatile toolkit to unlock their value for durable disease resistance breeding.

## De novo domestication

**Fig 2:**
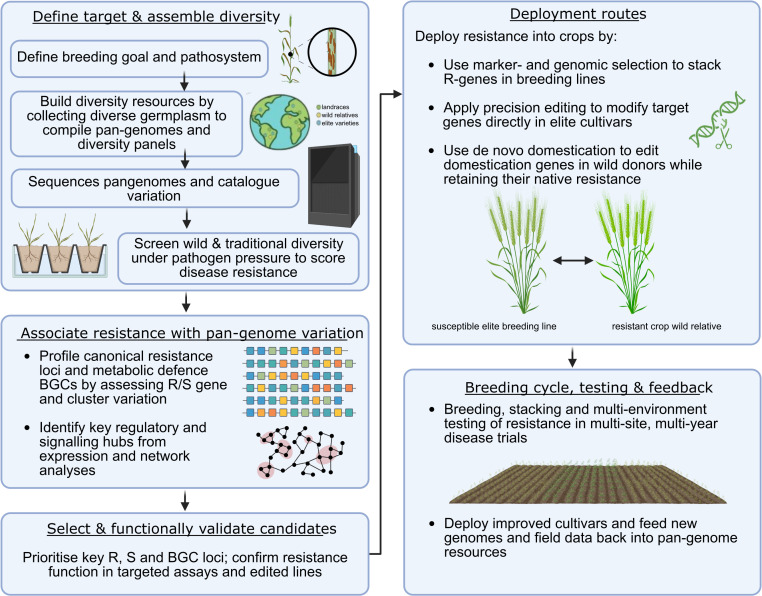
Integrating pan-genomes, wild relative screening and genome editing to develop improved disease-resistant crops. Schematic overview of how diversity resources, phenotyping, pan-genome analyses and functional validation inform breeding, precision editing and de novo domestication. Created in BioRender. Kamal, N. (2026) https://BioRender.com/4mtdik9.

Traditionally, *R* alleles from wild or locally adapted germplasm have been introgressed into elite cultivars to enhance disease resistance. A complementary strategy, de novo domestication, improves wild or underutilised species by editing key domestication genes while retaining their natural stress resilience [32]. Combining genome editing with pangenomic insight allows the transformation of hardy but non-domesticated plants into new crops adapted to future climates—preserving the complex resistance networks and metabolic defences that are often lost in elite lines (Fig 2).

Wild progenitors of cereals, legumes and solanaceous crops already contain alleles conferring tolerance to drought, heat and pathogens. Editing a small set of domestication genes controlling seed shattering, plant architecture or flowering can rapidly convert these wild relatives into cultivable, stress-tolerant crops. Proof-of-concept studies in tomato [19,33] and rice [34] demonstrate the feasibility of this strategy.

As genomic and functional data expand across wild species, de novo domestication offers a route to diversify agriculture—broadening crop portfolios, distributing production risks and reducing dependence on a few global staples. Integrated with comparative genomics, it will help identify and improve promising wild taxa, paving the way for a new generation of disease-resistant and climate-resilient crops.

## Conclusion and future perspectives

The explosion of sequencing data, expanding pan-genomes, and high-resolution multi-omics has opened unprecedented access to the genetic and metabolic diversity underpinning plant immunity. These resources now enable the systematic connection of allelic diversity found in landraces, crop wild relatives and pangenomes to resistance traits in crops, revealing structural variants and pathways that were previously hidden from traditional approaches. By integrating these datasets, we can move from cataloguing diversity to understanding its function and using it predictively.

The next phase of crop improvement will build on traditional breeding, which has long achieved remarkable progress through large-scale phenotypic and marker-assisted selection. Genomics and bioinformatics now extend this success by linking phenotypes to their causal variants, accelerating the discovery of durable resistance genes and guiding the introgression or editing of beneficial alleles. Establishing robust genotype-to-phenotype relationships is key to turning genomic diversity into lasting impact—revealing the mechanisms that underlie durable resistance, informing the deployment of alleles that remain effective across environments and stabilising yield under a changing climate.

Decades of breeding and association studies have uncovered extensive genetic variation underlying resistance traits. Integrating functional assays and mechanistic analyses with these genomic resources helps explain why particular alleles confer durable resistance, how they interact with signalling and metabolic pathways, and under which environmental conditions they remain effective. This mechanistic understanding enables the design of predictive markers and the rational combination of stacked or complementary resistance mechanisms, extending the durability and stability of resistance already achieved through breeding.

Future efforts should expand exploration of global genetic diversity—across wild progenitors, landraces and underrepresented genebanks—while strengthening local capacity to conserve and study it (Fig 2). Equitable partnerships and training, including smaller institutions and developing regions, are essential to ensure diversity is used collaboratively rather than selectively. Integrating discovery with breeding practice across scales and regions will enable the development of climate-resilient, disease-resistant cultivars that stabilise yields, support ecosystems, and empower communities in crop improvement.

## Supporting information

S1 Table Immune-related genes and gene families discussed in the section “Pan-genomics uncovers hidden structural variants associated with immunity” and highlighted in Fig 1, with abbreviations, full gene names, brief functional descriptions, and representative gene identifiers in *Arabidopsis thaliana* and Solanum [19] that were identified as expanded in wild vs. domesticated species. (XLSX)
